# The Use of Response Surface Methodology as a Statistical Tool for Media Optimization in Lipase Production from the Dairy Effluent Isolate *Fusarium solani*

**DOI:** 10.5402/2013/528708

**Published:** 2012-10-22

**Authors:** P. Kanmani, S. Karthik, J. Aravind, K. Kumaresan

**Affiliations:** Department of Biotechnology, Kumaraguru College of Technology, Coimbatore 641049, India

## Abstract

The optimization of extracellular lipase production by *Fusarium isolani* strain SKWF7 isolated from dairy wastewater was carried out in this study. Initially, the physicochemical factors significantly influencing enzyme production were studied by varying one-factor-at-a-time (OFAT). A mesophilic temperature of 40°C, alkaline pH of 8, and incubation period of 72 hours were found to be the optimal conditions for lipase production. Among the media components, the disaccharide sucrose acted as the best carbon source; palm oil as the best inducing lipid substrate; casein and (NH_4_)_2_SO_4_ as the best organic and inorganic nitrogen sources; Ca^2+^ ion as the best trace element. In the next phase of work, statistical optimization of medium components was performed by employing the Box-Behnken design of Response Surface Methodology (RSM). The optimum concentrations of three significant factors, namely, palm oil, (NH_4_)_2_SO_4_, and CaCO_3_ were determined by this method to be 5% (v/v), 5.5 g/L, and 0.1 g/L, respectively. RSM-guided design of experiments resulted in a maximum lipase production of 73.3 U/ml, which is a 1.7-fold increase in comparison with that obtained in the unoptimized medium. These results point towards the success of the model in developing a process for the production of lipase, an enzyme of enormous industrial significance.

## 1. Introduction

Lipases are enzymes that belong to the class of hydrolases and are involved in catalyzing the hydrolysis of triglycerides to fatty acids and glycerol, this reaction occurs at the oil-water interface [1]. Besides, they are also capable of catalyzing the reverse reaction, that is, ester synthesis, in water-restricted environments [2]. Transesterification and resolution of racemic mixtures are also reactions that could be facilitated by lipases. Such a versatile nature has paved the way for their application in diversified industries including food, dairy, cosmetic, detergent, and pharmaceuticals [3]. The environmental applications of the enzyme are also innumerable, where they break down fat, oil, and greasy material in the wastewater [4]. These constituents could pose problems in the sewers as well as the treatment plant, impeding oxygen transfer in aerobic biological treatment systems. These are some of the underlying reasons for a sustained interest in the enzyme.

Lipases can be procured from plant, animal [5–8], and microbial sources. However, microbial lipases have gained increasing attention due to their stability and high substrate specificity [9, 10]. In the microbial community, lipolytic activity is exhibited by several bacteria, fungi, yeasts, and among these, filamentous fungi are the ideal candidates for industrial-scale lipase production [11]. *Aspergillus, Penicillium, Mucor, Rhizopus, Geotrichum*, and so forth are some of the fungal genera whose lipase activities have been widely investigated [11–13].

New organisms continue to be screened, with hopes of stumbling upon isolates with extremophilic properties that can survive harsh conditions and produce enzymes with unique characteristics desirable for specific industrial applications. After this initial step of isolation and screening, optimization of enzyme production by the isolate is warranted. pH, temperature, aeration rate, incubation period, inoculum level and age, carbon, nitrogen sources and trace elements in the medium, and so forth are some of the physicochemical factors that influence enzyme production and these have to be optimized [14].

Medium optimization has conventionally been done by varying one factor at a time (OFAT), and it is workable as long as the production process is influenced by a limited number of variables. Even then, it fails to assess the interaction effects of those variables and becomes quite cumbersome, entailing a large number of trials when several variables are to be considered. Hence, researchers seek the aid of statistical tools such as Placket Burman and Response Surface Methodology (RSM) for effective optimization of their production media [15, 16]. This applies to lipase production as well [17]. 

In our study, fungal strains isolated from dairy wastewater were screened for lipase activity; physicochemical parameters having significant impact on lipase production were identified through the classical OFAT approach, and later RSM (Box-Behnken Design) was applied in determining the optimal concentrations as well as interaction effects of those parameters.

## 2. Materials and Methods

### 2.1. Chemicals

p-nitrophenyl palmitate (p-NPP) was purchased from Sigma Aldrich, USA. All other chemicals and reagents used were of analytical grade and purchased from Hi-Media, India.

### 2.2. Sample Collection

Wastewater sample was collected from “Central Ghee” dairy industry, Gobichettipalayam, Tamil Nadu, India. It was stored at 4°C and used as a source of possible lipolytic fungal strains.

### 2.3. Isolation and Screening

The effluent sample was subjected to serial dilution and then plated on potato dextrose agar (PDA) plates for fungal isolation. Colonies on the plate that showed morphological difference were subcultured on PDA slants and maintained as pure culture. Such pure cultures were inoculated into glucose-yeast extract-peptone (GYP) broth supplemented with 2% olive oil and containing trace amounts of mineral salts (glucose 20 g/L, yeast extract 10 g/L, peptone 10 g/L, CH_3_COONa·3H_2_O 10 g/L, MgSO_4_ 0.3 g/L, MnSO_4_ 0.1 g/L, CuSO_4_·5H_2_O 1.5 mg/L, KCl 0.5 g/L). They were cultured at room temperature for 48 hours in a rotary shaker (90 rpm) after which they were centrifuged at 10,000 g for 10 minutes at 4°C. The supernatant was used as the crude enzyme extract, whose activity was assayed by using p-NPP as substrate (ELICO UV-VIS Spectrophotometer) [18]. 

For the enzyme assay, 30 mg of p-NPP was dissolved in 10 mL of isopropanol (solution A); 0.1 g of gum arabic and 0.4 mL Triton X-100 were added to 90 mL of 50 mM Tris-HCl buffer, pH 8 (solution B). They were mixed together to get the substrate solution. To 9 mL of this solution, 1 mL of enzyme was added and the mixture incubated at 32 ± 2°C for 15 min. Its absorbance was measured at 410 nm, the absorption coefficient of p-nitrophenol at 410 nm is 0.0148. One unit of lipase activity (U) was expressed as *μ*mol of p-nitrophenol released per minute under the assay conditions. The isolate that exhibited maximum activity was selected for further optimization studies. 

### 2.4. Optimization of Production Medium 

#### 2.4.1. OFAT Method

Growth conditions and chemical components were varied one at a time in the GYP medium and their impacts on lipase production were studied. The incubation time of the culture was varied in the range of 12–96 hours; the incubation temperature in the range of 30–60°C; initial pH in the range of 4.0–12.0. The chemical composition of the growth medium was then altered in the following ways: glucose was substituted with starch, sucrose, or maltose (2%); nitrogen source was replaced by other organic (1%) or inorganic (0.5%) sources, namely, soy peptone, casein, sodium nitrate, ammonium sulfate, or ammonium chloride; olive oil was replaced with gingelly, sunflower, groundnut, palm, or tributyrin oil (2%); finally, the basic GYP medium was augmented with trace amounts of different inorganic ions (Ca^2+^, Fe^3+^, Zn^2+^, Ba^2+^, or Mg^2+^).

#### 2.4.2. Response Surface Methodology

The interactive effects of three significant factors *A* (oil source), *B* (nitrogen source), and *C* (inorganic ion) on the response, namely, lipase production were determined statistically using RSM. A Box-Behnken design developed by the Design Expert software, version 8.0.7.1 (Stat Ease Inc. Minneapolis, USA, trial version) was adopted for this purpose.

Each one of the above independent variables *A*, *B*, and *C* was taken at a central coded value considered as zero and studied at three different levels. The values of these variables in actual and coded forms are presented in Table 1. A matrix consisting of 17 experiments with 5 replicates at the centre point generated by the software was applied for maximizing the lipase production. Production was carried out in 250 mL Erlenmeyer flasks containing 100 mL of the production medium (pH 8.0). The flasks were sterilized by autoclaving at 120°C for 20 min, inoculated with the culture under aseptic conditions, and incubated at 40°C for 72 h, in an orbital shaker set at 90 rpm. At the end of the incubation period, lipase assay was performed using the cell-free supernatant and activity was recorded as the response (dependent variable). All experiments were carried out in triplicate and the data represent the mean. The following second-order polynomial equation describes the relationship between the dependent and independent variables:

(1) Y=β0+β1A+β2B+β3C+β11A2+β22B2+β33C2+β12AB+β13AC+β23BC,

where, *Y* is the predicted response, *β*_0_ is the intercept, *β*_1_, *β*_2_, *β*_3_, are the linear coefficients, *β*_11_,  *β*_22_,  *β*_33_, are the squared coefficients, and *β*_12_, *β*_13_, *β*_23_ are the interaction coefficients.

The model was statistically analyzed. Analysis of variance (ANOVA) involved Fischer's *F* test to judge the model's overall significance, associated probability values, and coefficient of determination to measure the regression model's goodness of fit. The fitted polynomial equation was further expressed in the form of 3D and contour plots which depicted the interactions graphically. Finally, the model was also validated.

## 3. Results and Discussion

### 3.1. Isolation and Screening

**Figure 1:**
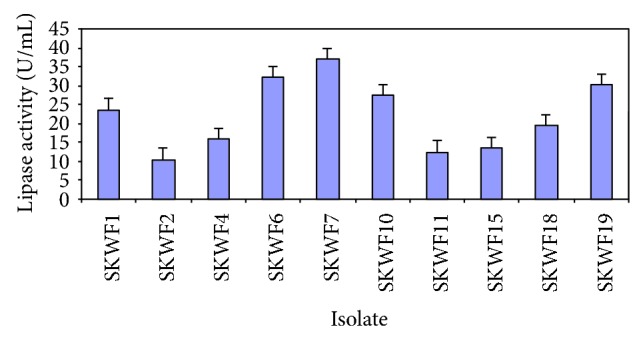
Lipase activities of different fungal isolates.

Several fungal colonies grew on the PDA plates that were inoculated with the serially diluted wastewater sample. 19 morphologically different colonies were subcultured and the resulting pure cultures were preserved on PDA slants overlaid with liquid paraffin. p-NPP assay of these isolates after cultivation in the production medium revealed appreciable activities for 10 (Figure 1) and negligible activities for the rest (data not shown). The isolate SKWF7, which showed a maximum activity of 36.9 U/mL was identified to be *Fusarium solani* by the Indian Agricultural Research Institute (IARI), New Delhi. Further experiments on medium optimization were performed with this isolate.

### 3.2. Optimization of Production Medium: OFAT Method

When the physicochemical parameters were varied one at a time and their impact on lipase production was investigated, the following results were obtained.

**Figure 2:**
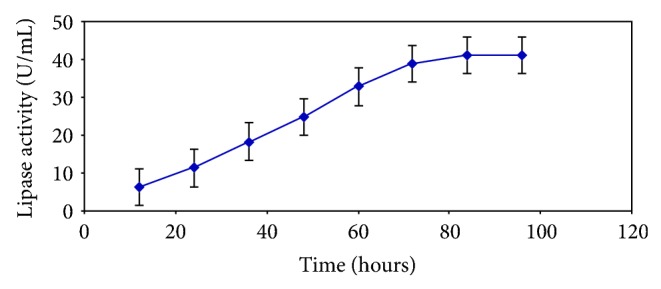
Effect of incubation time on lipase production.

**Figure 3:**
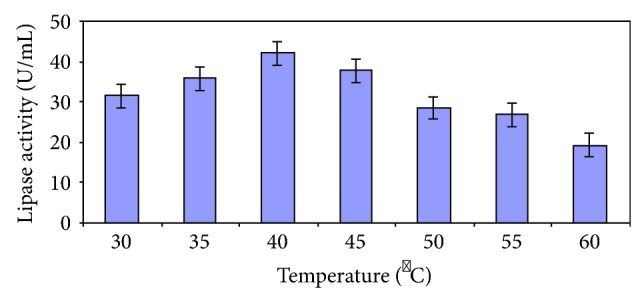
Effect of temperature on lipase production.

**Figure 4:**
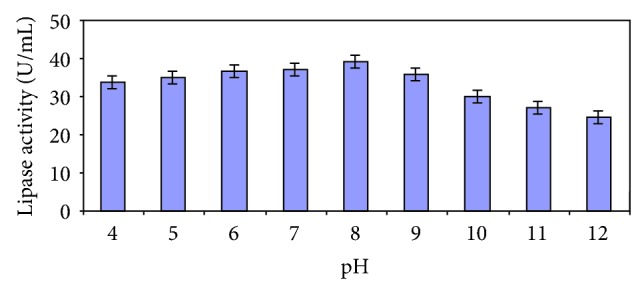
Effect of pH on lipase production.

Enzyme production steadily increased with increasing incubation time of the culture and reached a plateau at 72 hours (Figure 2). Similar or longer incubation periods have been widely reported for fungi [19]. In the case of *Mucor* sp., highest production was obtained only after 6 days of cultivation [20]. Highest enzyme production was recorded with an incubation temperature of 40°C (Figure 3). Upon altering the culture medium pH, it was witnessed that there was good production in the range of 4.0–8.0, with the production peaking at 8 and rapidly falling thereafter (Figure 4). A strain of *Aspergillus niger* showed maximum enzyme production after 96 h of growth in a medium with an initial pH of 7.0 [21].

**Figure 5:**
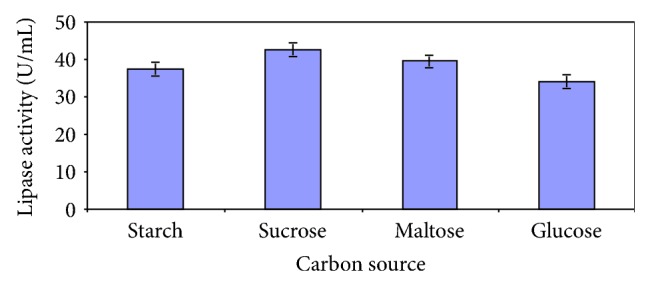
Effect of carbon source on lipase production.

**Figure 6:**
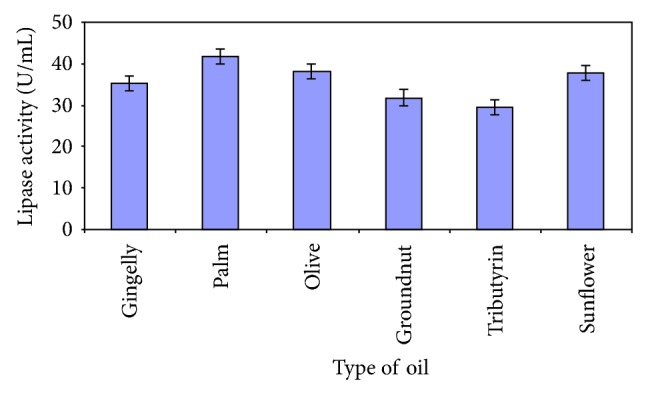
Effect of oil source on lipase production.

The disaccharide sucrose was found to be the best carbon source (Figure 5). The inducible nature of lipases necessitates the presence of a lipid substrate in the medium and in this case, induction of enzyme synthesis was best favoured by the presence of palm oil (Figure 6). A combination of 2% olive oil and 2% glucose was demonstrated to be conducive for lipase production by *A. niger* [22].

**Figure 7:**
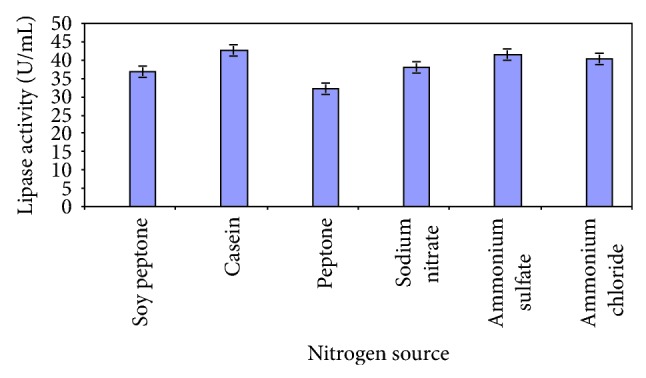
Effect of nitrogen on lipase production.

The organic nitrogen source casein and inorganic source ammonium sulphate resulted in maximum lipase production (Figure 7). *Fusarium oxysporum *showed optimal production with the use of peptone and ammonium dihydrogen phosphate [23].

**Figure 8:**
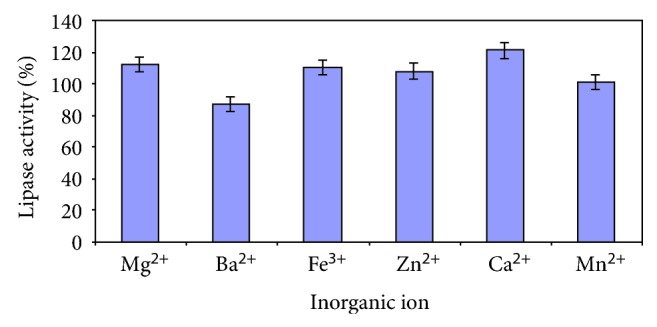
Effect of inorganic ions on lipase production.

Ca^2+^ best stimulated the enzyme production by up to 20%, in comparison to a medium devoid of trace elements (Figure 8). Several reports vouch for the stimulatory role of this element [24]. The presence of Mn^2+^ had no effect, while Ba^2+^ had a negative impact on enzyme production.

### 3.3. Response Surface Methodology

RSM incorporates the interaction effects of variables and aids us in simultaneously optimizing several process parameters within a minimal number of experimental runs. Such statistically assisted experimental designs can lead to significantly enhanced production. In our investigation, palm oil (*A*), (NH_4_)_2_SO_4_ (*B*) and CaCO_3_ (*C*), which were inferred to be significant media constituents based on OFAT studies were selected for optimization through the Box-Behnken design. The experimental design set up is shown in Table 1.

Second-order polynomial equation describing the empirical relationship between the independent variables and response is given underneath:

(2) Y(lipase  activity U/mL)=+67.20527−19.48972A  +3.82302B−36.39877C  +0.11778AB−0.030556AC  −0.12840BC+3.73469A2  −0.34093B2+30.19136C2.

The ANOVA results and diagnostic case studies are given in Tables 2 and 3, respectively. The *P* value serves as a tool for checking the significance of each of the coefficients and is indicative of the interaction strength of each independent variable. Low values of *P* < 0.05 indicate high significance of the corresponding coefficients. In general, larger *t*, *F* and smaller *P* values indicate that the corresponding coefficient terms are significant [25]. Subjecting our model to ANOVA showed an *F* value of 1704.58 and a *P* value < 0.0001 which indicate that the model is highly significant. All three linear coefficients, squared coefficients, and one interaction coefficient (*AB*) are significant, as evidenced from low *P* and high *F* values.

*R* ^2^ value gives a measure of how much variability in the observed response can be explained by the experimental parameters and their interactions [26]. The *R* ^2^ value for this model is 0.9995. When expressed as a percentage, it implies that a total variation of 99.95% in enzyme activity can be attributed to the independent variables and only 0.05% cannot be ascribed to them. The predicted *R* ^2^ of 0.9927 is in acceptable agreement with the adjusted *R* ^2^ of 0.9989. The adjusted *R* arranges the *R* values for the sample size and for the number of variables in the model. 

Adequate precision measures signal-to-noise ratio and detects which experimental parameters generate signals that are large in comparison to the noise. A ratio greater than 4 is desirable. In this case, the value obtained is 327.78 and is thus indicative of an ample signal. 

**Figure 9:**
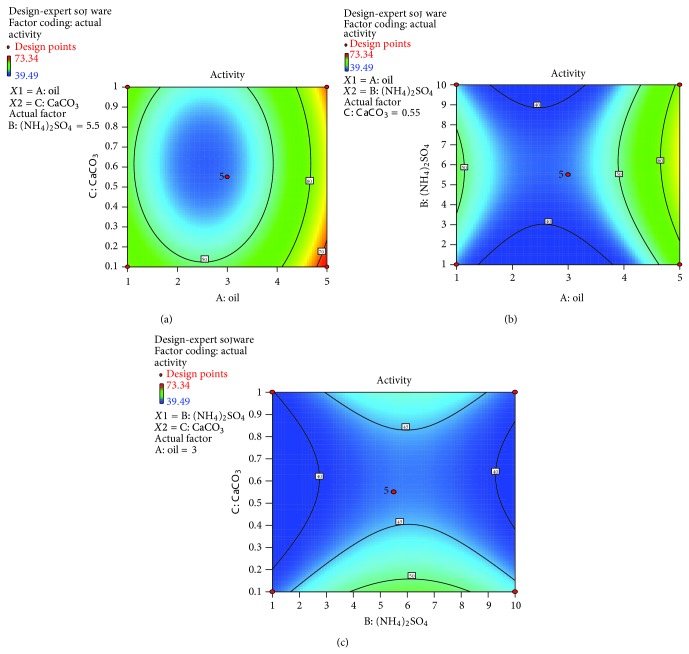
(a) Plot between effects of palm oil and calcium carbonate on lipase production, (b) plot between effects of palm oil and ammonium sulfate on lipase production, (c) plot between effects of ammonium sulfate and calcium carbonate on lipase production.

The regression equation is expressed graphically in the form of contour plots which depict the interactions among the independent variables and their influence on enzyme production. The contour plots might be elliptical mounds, saddle points, or rising ridges [27]. Here, contour plots were generated by varying the levels of two factors while keeping the third one constant. The plot of oil concentration versus calcium carbonate concentration (Figure 9(a)) is elliptical in nature indicating significant interactions between the two variables. The plot between (NH_4_)_2_SO_4_ and oil concentrations Figure 9(b) and the one between (NH_4_)_2_SO_4_ and CaCO_3_ concentrations Figure 9(c) are not elliptical in nature, but saddle points, indicating that there are fewer interactions between them.

RSM design yielded a maximum lipase activity of 73.3 U/mL. This was obtained in trial no. 13 and the levels of the independent variables were 5% oil (+1), 5.5 g/L (NH_4_)_2_SO_4_ (0), and 0.1 g/L CaCO_3_ (−1). This is a 1.7-fold increase in comparison with the preoptimization maximum of 42.3 U. This goes to prove that the developed model has resulted in appreciable enhancement of enzyme production, thereby improving the feasibility of the process. Earlier, central composite design has been applied to optimize lipase production by the yeast *Candida cylindracea* using two different carbon sources and a yield of 17.30 U/mL using glucose as carbon source and 47.25 U/mL using olive oil as carbon source was obtained [27]. Lipase production by *Candida rugosa* has been optimized using RSM-based design and cheese whey was used as a substrate for this. From the experimental design it was concluded that a combination of brewery product, yeast and malt extract, Tween 80, and olive oil with cheese whey resulted in a 287% increase in enzymatic activity [28]. A medium comprised of soyabean meal 0.77% (w/v), (NH_4_)_2_SO_4_ 0.1 M, KH_2_PO_4_ 0.05 M, rice bran oil 2% (v/v) CaCl_2_ 0.05 M, PEG 6000–0.05% (w/v), NaCl 1% (w/v), inoculum 1% (v/v), pH 3, incubation temperature 35°C, and incubation period of 5 days has been identified to be the optimal conditions for lipase production by *Aspergillus awamori *[29]. In another study, a medium containing 0.45% (w/v) peptone, 0.65% (v/v) Tween-80, and 2.2% (v/v) inoculum has been recommended for a maximal lipase production of 20.6 U/mL by *Candida cylindracea *[30]*.*

#### 3.3.1. Model Verification and Confirmation

The objective of the validation study is to demonstrate that the polynomial expression could correctly predict and describe the response function. The validation report generated by the design expert software is given in Table 4, which predicts a maximum lipase activity of 73.65 U/mL.

## 4. Conclusion

This work has culminated in the isolation of several fungal strains from dairy effluent, of which *Fusarium solani* SKWF7 exhibited pronounced extracellular lipolytic activity. The physical and chemical parameters influencing enzyme production by this isolate were varied one at a time and their impact was probed, revealing the ones that lead to a heightened enzyme production. The feasibility of using statistically based experimental design to simultaneously screen multiple variables and optimize fermentation conditions for increasing the enzyme yield was also demonstrated. Based on this design, 5% (v/v), 5.5 g/L, and 0.1 g/L were found to be the optimal concentrations of palm oil, (NH_4_)_2_SO_4_, and CaCO_3_, resulting in a 1.7-fold increase in enzyme production. The model was statistically significant as is evident from low *P* and high *F* values. Thus, it can be concluded that the model can potentially be applied for lipase production by *Fusarium solani.* Based on the promising results obtained in this shake-flask study, production in a bioreactor and the utilization of cheap substrates derived from waste materials are to be taken up in future studies, with an objective of exploring scale-up feasibility, bringing down production costs and thereby augmenting the economic viability of the process. 

## Figures and Tables

**Table 1:** Experimental design of Box-Behnken.

Std	Trial	Oil (%v/v)	(NH_4_)_2_SO_4_ (g/L)	CaCO_3_ (g/L)	Lipase (U)
15	1	3 (0)	5.5 (0)	0.55 (0)	43.68
10	2	3 (0)	10 (+1)	0.1 (−1)	46.81
2	3	5 (+1)	1 (−1)	0.55 (0)	56.11
1	4	1 (−1)	1 (−1)	0.55 (0)	44.03
12	5	3 (0)	10 (+1)	1 (+1)	42.08
7	6	1 (−1)	5.5 (0)	1 (+1)	56.18
11	7	3 (0)	1 (−1)	1 (+1)	39.49
8	8	5 (+1)	5.5 (0)	1 (+1)	70.32
3	9	1 (−1)	10 (+1)	0.55 (0)	45.2
9	10	3 (0)	1 (−1)	0.1 (−1)	43.18
16	11	3 (0)	5.5 (0)	0.55 (0)	43.68
13	12	3 (0)	5.5 (0)	0.55 (0)	43.68
6	13	5 (+1)	5.5 (0)	0.1 (−1)	73.34
14	14	3 (0)	5.5 (0)	0.55 (0)	43.68
5	15	1 (−1)	5.5 (0)	0.1 (−1)	59.09
4	16	5 (+1)	10 (+1)	0.55 (0)	61.52
17	17	3 (0)	5.5 (0)	0.55 (0)	43.68

(+1), (0) and (−1) are coded values.

**Table 2:** ANOVA for response surface quadratic model.

Source	Sum of squares	df	Mean square	*F* value	*P* value Prob > *F*
Model	1734.05	9	192.67	1704.58	<0.0001
*A*-Oil	403.14	1	403.14	3566.58	<0.0001
*B*-(NH_4_)_2_SO_4_	20.48	1	20.48	181.19	<0.0001
*C*-CaCO_3_	25.74	1	25.74	227.73	<0.0001
*AB*	4.49	1	4.49	39.76	0.0004
*AC*	3.03*E* − 003	1	3.025*E* − 003	0.027	0.8747
*BC*	0.27	1	0.27	2.39	0.1659
*A* ^ 2^	939.65	1	939.65	8313.10	<0.0001
*B* ^ 2^	200.68	1	200.68	1775.43	<0.0001
*C* ^ 2^	157.38	1	157.38	1392.35	<0.0001
Residual	0.79	7	0.11		
Lack of fit	0.79	3	0.26		
Pure error	0.000	4	0.000		
Cor total	1734.84	16			

Predicted *R* ^2^: 0.9927 adjusted *R* ^2^: 0.9989 adequate precision: 327.78.

**Table 3:** Diagnostics case statistics.

Standard order	Actual value	Predicted value	*t*-test values	Run order
1	44.03	44.08	−0.275	4
2	56.11	56.15	−0.260	3
3	45.20	45.16	0.260	9
4	61.52	61.47	0.275	16
5	59.09	59.40	−1.844	15
6	73.34	73.65	−1.859	13
7	56.18	55.87	1.859	6
8	70.32	70.01	1.844	8
9	43.18	42.82	2.119	10
10	46.81	46.54	1.584	2
11	39.49	39.76	−1.584	7
12	42.08	42.44	−2.119	5
13	43.68	43.68	0.000	12
14	43.68	43.68	0.000	14
15	43.68	43.68	0.000	1
16	43.68	43.68	0.000	11
17	43.68	43.68	0.000	17

**Table 4:** Model verification and confirmation.

Factor	Name	Level	Low level	High level	Std. Dev.
*A*	Oil	5	1	5	0
*B*	(NH_4_)_2_SO_4_	5.5	1	10	0
*C*	CaCO_3_	0.1	0.1	1	0
Response	Prediction	Std. Dev.	SE (*n* = 1)	95% PI low	95% PI high
Activity	73.652	0.336	0.444	72.600	74.704
